# Prospective evaluation of the role of imaging techniques and TMPRSS2:ERG mutation for the diagnosis of clinically significant prostate cancer

**DOI:** 10.3389/fonc.2022.968384

**Published:** 2022-09-06

**Authors:** Massimo Lazzeri, Vittorio Fasulo, Giovanni Lughezzani, Alessio Benetti, Giulia Soldà, Rosanna Asselta, Ilaria De Simone, Marco Paciotti, Pier Paolo Avolio, Roberto Contieri, Cesare Saitta, Alberto Saita, Rodolfo Hurle, Giorgio Guazzoni, Nicolò Maria Buffi, Paolo Casale

**Affiliations:** ^1^ Department of Urology, Istituti di Ricovero e Cura a Carattere Scientifico (IRCCS) Humanitas Research Hospital, Milan, Italy; ^2^ Department of Biomedical Sciences, Humanitas University, Milan, Italy

**Keywords:** TMPRSS2:ERG, prostate cancer, mpMRI, microUS, gene fusion, translocation, diagnosis

## Abstract

**Objectives:**

To test the hypothesis of a relationship between a specific genetic lesion (T2:ERG) and imaging scores, such as PI-RADS and PRI-MUS, and to test the effectiveness of these parameters for the diagnosis of prostate cancer (PCa) and clinically significant PCa (csPCa).

**Materials and methods:**

This is a prospective study of men with suspected PCa enrolled between 2016 and 2019 at a high-volume tertiary hospital. Patients underwent systematic US-guided biopsy, plus targeted biopsy if they were presenting with >=1 suspicious lesion (PI-RADS>2) at mpMRI or PR-IMUS >2 at micro-ultrasound assessment. For each patient, one core from the highest PI-RADS or PRI-MUS lesion was collected for T2:ERG analysis. Multivariable logistic regression models (LRMs) were fitted for csPCa with a clinical model (age, total PSA, previous biopsy, family history for PCa), a clinical plus PI-RADS, clinical plus T2:ERG, clinical plus PI-RADS plus T2:ERG, and T2:ERG plus PI-RADS alone.

**Results:**

The cohort consists of 158 patients: 83.5% and 66.2% had respectively a diagnosis of PCa and csPCa after biopsy. A T2:ERG fusion was found in 37 men and 97.3% of these patients harbored PCa, while 81.1% were diagnosed with csPCa. SE of T2:ERG assay for csPCa was 28.8%, SP 87.0%, NPV 38.8%, and PPV 81.1%. Of 105 patients who performed mpMRI 93.% had PIRADS ≥3. SE of mpMRI for csPCa was 98.5%, SP was 12.8%, NPV was 83.3%, and PPV was 65.7%. Among 67 patients who were subjected to micro-US, 90% had a PRI-MUS ≥3. SE of micro-US for csPCa was 89.1%, SP was 9.52%, NPV was 28.6%, and PPV was 68.3%. At univariable LRM T2:ERG was confirmed as independent of mpMRI and micro-US result (OR 1.49, p=0.133 and OR 1.82, p=0.592, respectively). At multivariable LRM the clinical model alone had an AUC for csPCa of 0.74 while the clinical model including PI-RADS and T2:ERG achieved an AUC of 0.83.

**Conclusions:**

T2:ERG translocation and imaging results are independent of each other, but both are related csPCa. To evaluate the best diagnostic work-up for PCa and csPCa detection, all available tools (T2:ERG detection and imaging techniques) should be employed together as they appear to have a complementary role.

## Introduction

In 1992 prostate specific antigen (PSA) was introduced for diagnosis of prostate cancer (PCa). However, due to its poor specificity for clinically significant PCa (csPCa), the use of PSA as a standalone test still leads to diagnosis of many indolent tumors (1–3). The ideal biomarker should be acceptable to the patient, non-invasive, accurate, specific, cost-effective and should guide management decision and therapy (4). For this reason, lots of efforts have been made to find novel and alternative biomarkers (5–7). Nowadays, the panel of biomarkers available spreads from PSA derivates to genetic testing, which, in the light of all the recent advances, should be considered as an important part of diagnostic evaluation (5–7). As part of genetic testing, the transmembrane protease serine 2 and erythroblastosis virus E26 oncogene homolog (*TMPRSS2:ERG*) gene fusion has been studied as a PCa-specific biomarker since 2005 (8, 9). The *TMPRSS2:ERG* gene fusion (*T2:ERG*) is found in up to 50% of PCa and results in androgen-dependent overexpression of *ERG*, which has been demonstrated to be a key regulator of differentiation, apoptosis, embryonic development, cell proliferation, and inflammation (8). Therefore, early identification of such gene fusion may be helpful for an optimal management of patients. The identification of *T2:ERG* has been attempted in different specimens, such as peripheral blood, prostate tissue, seminal fluid, and urine (10–12). Since urine sample collection is non-invasive, simple, and cheap, the detection of *T2:ERG* in urine has been increasing over time (13). The detection of T2: ERG in the urine sample has been extensively validated, including as an association with PCA3 (14, 15).

On the other hand, in recent decades, imaging has also been used for detection of PCa and multiparametric magnetic resonance imaging (mpMRI) has become the preferred method, allowing for high quality visualization of the prostate and identification of PCa (16, 17).

For this reason, both the American College of Radiology (ACR) and the European Society of Uroradiology (ESUR) proposed the Prostate Imaging Reporting and Data System (PI-RADS) score with the intent to standardize prostate MRI (18, 19). Thus, currently mpMRI is mandatory in diagnostic approach of PCa in biopsy naive men and with prior negative biopsy findings, as per European Association of Urology (EAU) guidelines (20). Moreover, advances in technology lead to the development of other imaging approaches, such as the micro-ultrasound system (ExactVu™), a high-frequency (29 MHz) transrectal ultrasound device suggested by some authors as a more feasible, low-cost, and readily available option compared to mpMRI (21–23). As PI-RADS per mpMRI, the Prostate Risk Identification Using Micro-Ultrasound (PRI-MUS) risk identification protocol is the standard score when a micro-ultrasound is performed (24). Furthermore, the role of microUS and PET-PSMA in the diagnostic pathway of PCa has recently been evaluated, reinforcing the idea that the integration of different tools could be the silver bullet for the early diagnosis of csPCa (22, 25).

In addition to biomarkers and imaging, the use of risk calculators has been widely suggested by the authors and many of these risk calculators have the advantage of using easy to retrieve clinical variables and being freely accessible as a web tool/mobile application (23). Despite they utility, more accurate next-generation risk calculators including mpMRIs and biomarkers have been introduced and their usefulness has been demonstrated; the goal should always be to reduce the number of unnecessary missing biopsies by the least number of csPCa (7, 26).

Since the association between imaging and clinical results remains vague, this study aims to explore the hypothesis that a correlation between a specific genetic lesion (T2:ERG) and imaging scores, such as PI-RADS and PRI-MUS, exists, and may be useful for improving PCa and csPCa diagnosis.

## Material and methods

### Study population

This is a prospective cohort study of men admitted at a tertiary university hospital, for scheduled prostate biopsy, between 2017 and 2019. The study was approved by the local ethics committee (Prot. N. 1791, 06 June 2017). All patients signed a written informed consent. We included all patients (biopsy naïve, re-biopsy or reclassification biopsy on patient on active surveillance (AS) for PCa) referred to our institution for prostate biopsy who performed a genetic test to detect *T2:ERG* gene fusion. We excluded patient who were under hormonal therapy for PCa or who underwent radiotherapy for PCa. Type of imaging, biopsy decision and timing of the biopsy were at the discretion of the treating provider, patient preference.

All patients underwent systematic US-guided biopsy plus targeted biopsy only if they were presenting with ≥1 suspicious lesion (PI-RADS V.2 >2) at mpMRI or PRI-MUS >2 at micro-US assessment. Biopsies were either transrectal or transperineal depending on lesions’ location. Targeted mpMRI\TRUS biopsy has been performed with BioJet™ fusion system. For each patient, one prostatic core from the highest PI-RADS or PRI-MUS lesion was collected for *T2:ERG* analysis. In those patients with 2 or more lesions with the same score, the largest one was sampled. In those patients who had a negative mpMRI or micro-US, a core from the right lobe was collected as standard protocol and control. All histological analyses were performed by experienced genitourinary pathologists at our institution.

The primary endpoint was to evaluate the correlation between *T2:ERG* and PI-RADS/PRI-MUS scores. The secondary endpoint was to explore the additional value of T2:ERG next to imaging for the detection of csPCa.

### Acquisitions of mpMRI and micro-US data

All routine clinical mpMRI acquisitions were 1.5 Tesla, with endorectal coil, and 3.0 Tesla without endorectal coil. A dedicated experienced radiologist (>500 readings) reviewed all the mpMRIs. The PI-RADS v.2 score was used for this study (18, 19). All Micro-US were performed with a 29 MHz ExactVu™ transrectal micro-ultrasound system at our hospital by the most skilled urologists, M.L. and G.L.

### *T2*:*ERG* gene fusion detection

For RNA extractions, we used dedicated biopsy specimens, immediately immersed into the RNAlater stabilization solution (Sigma-Aldrich, St Louis, MO, USA) after withdrawal. RNA was extracted using the Maxwell RSC miRNA Tissue Kit (Promega, Madison, WI, USA) on a Maxwell RSC instrument (Promega). Random primers (Promega) and the Superscript-IV Reverse Transcriptase (Thermo Fisher Scientific, Waltham, MA, USA) were used to perform first-strand cDNA synthesis, following the manufacturer’s instructions. PCR reactions were accomplished starting from 2 µl of the reverse-transcription (RT) reaction and the GoTaq^®^ G2 Flexi DNA Polymerase (Promega). RT-PCRs were performed with different primer couples to detect the most frequent *T2:ERG* gene fusion events. To ensure the detection of *T2:ERG* fusion transcripts involving TMPRSS2 exon 1 and ERG exon 4, a nested PCR was performed by using 1.5 µl of the first PCR amplification, previously diluted 1:5. All amplified products were checked by direct Sanger sequencing using the BigDye Terminator Cycle Sequencing Ready Reaction Kit v1.1 (Thermo Fisher Scientific). Sequencing reactions were run on an ABI-3500 and analyzed using the FinchTV software. All oligonucleotides used in RT-PCR and sequencing steps were purchased from Sigma. Their sequences, as well as thermal profiles used for RT-PCRs, are available on request.

### Statistical analysis

Mean and standard deviation (SD) and median and interquartile ranges (IQRs) were used to compare normal or skewed continuous variables, respectively; frequencies were used for categorical variables. Demographic and clinical characteristics were compared by stratifying patients upon *T2:ERG* results. For 5 patients who performed biopsy and genetic test twice, the most recent results were considered for the analysis.

To test categorical variables “Chi-squared test of independence” and “Fisher exact test” were applied. To test categorical and continuous variable, ANOVA test or Kruskal-Wallis test were applied. We defined sensitivity (SE), specificity (SP), positive predictive value (PPV), and negative predictive value (NPV) of *T2:ERG* gene fusion, mpMRI and micro-US for detection of any PCa (grade group, GG ≥1) and csPCa, defined as GG ≥2. Positive mpMRI was defined as PI-RADS >2 and positive micro-US was defined as PRI-MUS >2.

Univariable logistic regression models (LRM) were performed to test the association with PCa or csPCa for the presence of *T2:ERG*, positive mpMRI, or positive micro-US. Furthermore, the univariable LRM was used to test the mpMRI and micro-US correlation with *T2:ERG*. Multivariable LRMs were fitted for csPCa with a clinical model (age, total PSA, previous biopsy, family history for PCa), a clinical plus PI-RADS, clinical plus *T2:ERG*, clinical plus PI-RADS plus *T2:ERG*, and *T2:ERG* plus PI-RADS alone. A model with PRI-MUS was not run due to the small sample size.

Statistical significance was set at p <0.05. Statistical analysis was done with STATA 16.1 (StataCorp, College Station, TX, USA).

## Results

### Baseline characteristics

A total of 158 patients, for whom a prostate core was collected, underwent a genetic test to detect *T2:ERG* gene fusion. Mean age was 66.4 ( ± 8.42) years old, while median PSA was 7.87 ng/ml (IQR 5.34-14.5). Family history was positive in 26 (16.5%) patients, 98 (62.0%) patients were biopsy naïve, 41 (25.9%) had a prior negative biopsy and 19 (12.0%) were on AS (**Table 1**).

**Table 1:** Baseline characteristics of the population.

Factor	Level	Value
**N**		158
**Age, mean (SD)**		66.4 (8.42)
**PSA, median (IQR)**		7.87 (5.37, 14.5)
**Family history, N (%)**	No	46 (29.1)
** **	Yes	26 (16.5)
** **	Unknown	86 (54.4)
**Prior biopsy, N (%)**	Naïve	98 (62.0)
** **	Repeated biopsy	60 (38.0)
**Active surveillance, N (%)**	No	139 (88.0)
** **	Yes	19 (12.0)

IQR, interquartile range; SD, standard deviation.

### Biopsy and genetic findings

Overall, a positive biopsy was recorded in 132 (83.5%) men: GG1 was found in 28 (21.2%) men, GG2 in 42 (31.8%), and ≥GG3 in 62 (47.0%). Median number of cores taken at each biopsy was 12 (IQR 10-14). In total, 34 (16.5%) were systematic and 124 (83.5%) were fusion biopsies (**Table 2**).

**Table 2:** Imaging and biopsy findings by absence or presence of T2:ERG translocation.

		Total	No translocation	TMPRSS2:ERG	p-value
		N=158	N=121	N=37	
**Family history, N (%)**	**No**	46 (29.1)	30 (24.8)	16 (43.2)	0.078
** **	**Yes**	26 (16.5)	22 (18.2)	4 (10.8)	
** **	**Unknown**	86 (54.4)	69 (57.0)	17 (45.9)	
**mpMRI result, N (%)**	**Negative**	6 (3.8)	6 (5.0)	0 (0.0)	0.17
** **	**Positive**	99 (62.7)	75 (62.0)	24 (64.9)	
** **	**Not performed**	53 (33.5)	40 (33.1)	13 (35.1)	
**PI-RADS, N (%)**	**1**	6 (3.8)	6 (5.0)	0 (0.0)	0.46
** **	**3**	29 (18.4)	23 (19.0)	6 (16.2)	
** **	**4**	42 (26.6)	32 (26.4)	10 (27.0)	
** **	**5**	27 (17.1)	19 (15.7)	8 (21.6)	
** **	**Not performed**	54 (34.2)	41 (33.9)	13 (35.1)	
**MICRO-US result, N (%)**	**Negative**	7 (4.4)	6 (5.0)	1 (2.7)	0.59
** **	**Positive**	60 (38.0)	46 (38.0)	14 (37.8)	
** **	**Not performed**	91 (57.6)	69 (57.0)	22 (59.5)	
**PRI-MUS, N (%)**	**1-2**	7 (4.4)	6 (5.0)	1 (2.7)	0.54
** **	**3**	5 (3.2)	4 (3.3)	1 (2.7)	
** **	**4**	34 (21.5)	28 (23.1)	6 (16.2)	
** **	**5**	21 (13.3)	14 (11.6)	7 (18.9)	
** **	**Not performed**	91 (57.6)	69 (57.0)	22 (59.5)	
**Type of biopsy, N (%)**	**Systematic**	34 (21.5)	26 (21.5)	8 (21.6)	0.99
** **	**Fusion**	124 (78.5)	95 (78.5)	29 (78.4)	
**Total # of core at biopsy, mean (±SD)**		12.0 (3.38)	12.1 (3.45)	12.0 (3.22)	0.89
**Total number of positive ROI, mean (±SD)**		1.89(1.77)	1.61 (1.74)	2.68 (1.63)	0.006
**Biopsy result, N (%)**	**Negative**	26 (16.5)	25 (20.7)	1 (2.70)	0.010
** **	**Positive**	132 (83.5)	96 (79.3)	36 (97.3)	
**ISUP, N (%)**	**1**	28 (17.7)	22 (18.2)	6 (16.2)	0.64
** **	**2**	42 (26.6)	29 (24.0)	13 (35.1)	
** **	**3**	25 (15.8)	19 (15.7)	6 (16.2)	
** **	**4**	18 (11.4)	11 (9.1)	7 (18.9)	
** **	**5**	19 (12.0)	15 (12.4)	4 (10.8)	
** **	**Negative**	26 (16.5)	25 (20.7)	1 (2.70)	
**Biopsy result csPCa, N (%)**	**Negative or LG**	54 (34.2)	47 (38.8)	7 (18.9)	0.025
	**csPCa**	104 (65.8)	74 (61.2)	30 (81.1)	

csPCa, clinically significant prostate cancer; ISUP, International Society of Urological Pathology; mpMRI, multiparametric Magnetic Resonance Imaging; PI-RADS, Prostate Imaging. Reporting and Data System; PRI-MUS, Prostate Risk Identification Using Micro-Ultrasound; SD, standard deviation; LG, low grade.

A *T2:ERG* fusion was found in 37 (23.4%) men and 36 (97.3%) had a positive biopsy (p=0.010): 6 (16.2%) with GG1, 13 (35.1%) with GG2 and 17 (45.9%) with ≥GG3. Of these patients, 29 (78.4%) underwent a fusion biopsy, of whom 96.4% had a positive target core and 90.9% had also a positive random one.

Among 127 (81%) men without the *T2:ERG* fusion, 26 (20%) had a negative biopsy versus 101 (80%) with positive one, 22 (17%) GG1, 31 (24%) GG2 and 48 (38%) ≥GG3.

**Figure 1:**
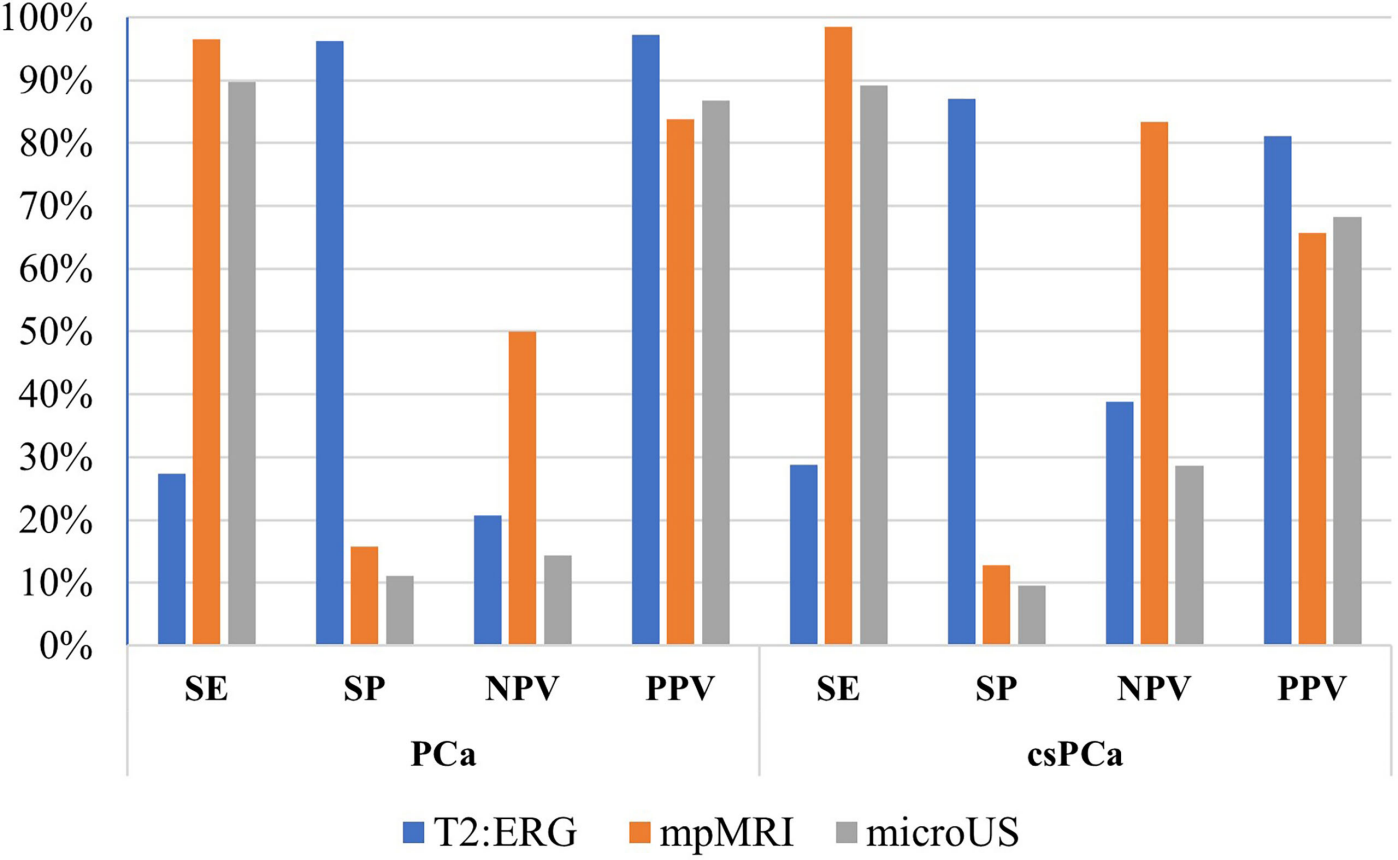
Diagnostic performance in comparison between fusion gene T2:ERG, mpMRI, and microUS.

SE of the *T2:ERG* assay for any PCa was 27.3% (95%CI 19.9-35.7), SP 96.2% (95%CI 80.4-99.9), NPV 20.7% (95%CI 13.8-29.0), and PPV 97.3% (95%CI 85.8-99.9). SE of T2:ERG assay for csPCa was 28.8% (95%CI 20.4-38.6), SP 87.0% (95CI% 75.1-94.6), NPV 38.8% (95%CI 30.1-48.1), and PPV 81.1% (95%CI 64.8-92.0) (**Figure 1**).

At univariable LRM, *T2:ERG* was associated with PCa (OR 9.37 95%CI 1.23-71.76, p= 0.031) and csPCa (OR 2.72 95%CI 1.11-6.69, p= 0.029).

### Imaging findings

Of 105 patients who performed mpMRI, 29 (27.6%) had a PI-RADS 3, while 69 (65.7%) had a PI-RADS 4-5. A fusion biopsy was performed in 95% of men with a PI-RADS ≥3. SE of the mpMRI for any PCa was 96.5% (95%CI 90.1-99.3), SP was 15.8% (95%CI 3.38-39.6), NPV was 50.0% (95%CI 11.8-88.2), and PPV was 83.8% (95%CI 75.1-90.5). SE of mpMRI for csPCa was 98.5% (95%CI 91.8-100), SP was 12.8% (95%CI 4.3-27.4), NPV was 83.3% (95%CI 35.9-99.6), and PPV was 65.7% (95%CI 55.4-74.9).

At univariable LRM mpMRI was not associated with PCa (OR 5.18 95%CI 0.96-28.1, p= 0.051) while it was with csPCa (OR 9.56 95%CI 1.07-85.1, p= 0.043). In addition there was an association of csPCa with PI-RADS 4 (OR 11.2 95%CI 1.18-105, p= 0.035) and PI-RADS 5 (OR 22 95%CI 2.08-232, p= 0.05)

Among 67 patients who were subjected to micro-US, 60 (90%) had a PRI-MUS 3-4-5, and 83% of these underwent a fusion biopsy. SE of micro-US for any PCa was 89.7% (95%CI 78.8-96.1), SP was 11.1% (95%CI 0.281-48.2), NPV was 14.3% (95%CI 0.361-57.9), and PPV was 86.7% (95%CI 75.4-94.1). SE of micro-US for csPCa was 89.1% (95%CI 76.4-96.4), SP was 9.52% (95%CI 1.17-30.4), NPV was 28.6% (95%CI 3.67-), and PPV was 68.3% (95%CI 55.0-79.7) (**Figure 1**).

At univariable LRM micro-US was not associated with PCa (p=0.944).

### *T2:ERG* correlation with imaging findings

All men carrying the *T2:ERG* fusion performed mpMRI and all had positive findings (PI-RADS>2), although the correlation was not significant (p=0.170). Of those with translocation who performed micro-US, 14/15 (93.3%) had positive results (PRI-MUS<2), but as for mpMRI there was no significant correlation (p=0.587).

At univariable LRM T2:ERG was confirmed as independent of mpMRI and micro-US result (OR 1.49 95%CI 0.886-2.50, p=0.133 and OR 1.82 95%CI 0.202-16.5, p=0.592, respectively).

At the multivariable LRM the clinical model alone had an AUC for csPCa of 0.74; adding PI-RADS or *T2:ERG* achieved an AUC of 0.84 or 0.78, respectively. The clinical model including PI-RADS and T2:ERG achieved an AUC of 0.83, and the AUC of a model with PI-RADS and T2:ERG was 0.73 (**Table 3**).

**Table 3:** Performance to multivariable logistic regression model of clinical variables only (age, total PSA, previous biopsy, family history for PCa) and clinical variables added by genetics, and imaging for the prediction of clinically significant PCa.

N° of patients	Model	AUC	95%CI	p-value
71	**Clinical**	0.74	0.61-0.86	0.0076
50	**Clinical + PI-RADS**	0.84	0.73-0.95	0.0104
71	**Clinical + T2:ERG**	0.78	0.67-0.88	0.0092
50	**Clinical + PI-RADS + T2:ERG**	0.83	0.72-0.94	0.0176
104	**PI-RADS + T2:ERG**	0.73	0.64-0.83	0.0008

AUC, Area under the ROC Curve; CI, confidence interval; PI-RADS, Prostate Imaging Reporting and Data System; T2:ERG, TMPRSS2:ERG.

### Radical prostatectomy findings

Forty-two men underwent radical prostatectomy: 14 (33.3%) with the *T2:ERG* fusion versus 28 (66.6%) without translocation (p=0.077). In patients with translocation, GG was ≥2 for all men, however we did not find a statistically significant difference in GG compared to those without translocation (p=0.870).

Lymphadenectomy dissection was performed in 39 men, and we found a positive finding in 5 men, of whom 2 with the translocation (p=0.840). Prostatectomy characteristics are summarized in **Table 4**.

**Table 4:** Radical prostatectomy findings by absence or presence of T2:ERG translocation.

		Total	No translocation	TMPRSS2:ERG	p-value
		N=42	N=28	N=14	
**ISUP, N (%)**	**1**	1 (2)	1 (4)	0 (0)	0.87
** **	**2**	18 (43)	11 (39)	7 (50)	
** **	**3**	17 (40)	12 (43)	5 (36)	
** **	**4**	2 (5)	1 (4)	1 (7)	
** **	**5**	4 (10)	3 (11)	1 (7)	
**pTNM, N (%)**	**pT2aN0**	6 (14)	4 (14)	2 (14)	0.78
** **	**pT2bN0**	1 (2)	1 (4)	0 (0)	
** **	**pT2cN0**	16 (38)	12 (43)	4 (29)	
** **	**pT2cN0R1**	1 (2)	1 (4)	0 (0)	
** **	**pT2cNx**	3 (7)	2 (7)	1 (7)	
** **	**pT3aN0**	4 (10)	1 (4)	3 (21)	
** **	**pT3aN0R1**	4 (10)	2 (7)	2 (14)	
** **	**pT3aN1R1**	2 (5)	1 (4)	1 (7)	
** **	**pT3aNx**	1 (2)	1 (4)	0 (0)	
** **	**pT3bN1R1**	3 (7)	2 (7)	1 (7)	
** **	**pT3bR1**	1 (2)	1 (4)	0 (0)	
**Margins, N (%)**	**Negative**	31 (74)	21 (75)	10 (71)	0.80
** **	**Positive**	11 (26)	7 (25)	4 (29)	
**Positive nodes, N (%)**	**Negative**	34 (87)	22 (88)	12 (86)	0.84
** **	**Positive**	5 (13)	3 (12)	2 (14)	
**Frozen section results, N (%)**	**Negative**	31 (86)	24 (96)	7 (64)	0.010
** **	**Positive**	5 (14)	1 (4)	4 (36)	

ISUP, International Society of Urological Pathology.

## Discussion

In this study, we observed a strong correlation between *T2:ERG* gene fusion and PCa, however we did not find an association of the gene mutation with mpMRI and/or micro-US. Those data lead speculating that *T2:ERG* gene fusion does not determine specific tissue rearrangement which could be specifically detected by MRI or micro-US.

The overexpression of the oncogene *ERG* in PCa was firstly described in 2005 (27). In the same period, its activation mechanism was discovered through fusion with *TMPRSS2*, driven by androgens that favor the overexpression of *ERG* (28, 29). In our study, the prevalence of *T2:ERG* was 23.4%, which is concordant with the range reported in the current literature, which varies from 7% to 83% according to different ethnic and geographic groups, as well as the type of analyzed material (e.g. biopsy, surgical specimen, urine) (30, 31).

A study by Mosquera et al. reports a high prevalence (46%) of translocation among 140 men diagnosed with PCa. Of note, all men with *T2:ERG* had cancer while no mutations were found in men with benign histological prostatic hyperplasia (31). As reported by Zhou et al. changes in the prevalence of T2: ERG may reflect racial differences (32).

Given its strong correlation with PCa and the possibility of isolating prostate cells or nucleic acids with the mutation on urine samples, *T2:ERG* has been proposed as a biomarker for PCa with rather low SE, 37%, but high SP, 93%, and PPV, 94% in post- digital rectal examination (DRE) urine specimens (33, 34). Similarly, we found a low SE for PCa, 27%, but high SP and PPV, 96% and 97%, respectively. The SE was low also for the detection of csPCa, 28%.

For these reasons, many authors combined the translocation with other biomarkers as screening/diagnostic tool (35). A study of Hessel et al. evaluated *T2:ERG* in association with Prostate Cancer Antigen 3 (PCA3) and reported a SE of 73%. Conversely, Stephan et al. reported an AUC of 0.63 for the gene fusion alone versus 0.74 for PCA3 alone, and interestingly the combination of both biomarkers did not result in a significant increase of the accuracy (36, 37). In our study, the AUC for PCa of *T2:ERG* alone was 0.62, but increased to 0.78 when combined with clinical data; moreover, adding at T2:ERG the mpMRI result the AUC for PCa reach 0.74

Recently, ExosomeDX, a biomarker deriving from the association of *T2:ERG* and PCA3, was evaluated in combination with mpMRI, demonstrating that this combination is an added value for screening to avoid unnecessary biopsies (7).

The association between mpMRI and PCa has been extensively studied and the association with csPCa is widely accepted (38, 39). Our results are in agreement with the literature: in fact, we found a significant (p=0.043) association with PI-RADS 4 and only a suggestive association signal with PI-RADS 5 and PCa, probably due to the small sample size.

As a new “easy to use” diagnostic tool, the micro-US has been proposed for the diagnosis and screening of PCa with an accuracy comparable to mpMRI, as confirmed by the Lughezzani and colleagues, which also showed PRI-MUS as an independent risk factor for PCa (21, 40). In this cohort, there was no association between micro-US and PCa, probably due to the small sample size (67 patients).

Here, we observed that *T2:ERG* is an independent risk factor compared to mpMRI and microUS. A possible explanation could be that, since the mutation is an early event during PCa tumorigenesis, it does not give signal alterations to mpMRI or typical echogenicity to microUS (41, 42). Likewise, specific histological patterns are not clearly detected by mpMRI, such as the cribriform pattern known to be associated with more aggressive disease (36, 37).

There are some limitations to be acknowledged. First, the sample size is relatively small and not all patients received a standardized diagnostic work-up. Additionally, the presence of *T2:ERG* was estimated from a bioptic sample and not from a urine sample so the possible detection could be underestimated (urine samples are representative of the entire gland, whereas biopsy does not reflect the heterogeneity of the disease).Moreover, a further limitation of the study could be a “selection bias” as the PI-RADS score on mpMRI is an indication for biopsy and could consequently be associated with T2: ERG. Furthermore, when we estimated the clinical model, due to the lack of complete volume values, we did not estimate the model added by PSA density, which is a well-known and crucial component for PCa risk prediction.

With the development of precision medicine, the association of *T2:ERG* with germline mutations is arousing more and more interest: in the era of selective and targeted medicine with dedicated screening pathways, PCa management should include specific machine learning algorithms implemented with the detection of specific gene mutations (43, 44).

However, large-scale studies are needed to identify the role of *T2:ERG* in real-world clinical practice and its role as a possible target for a future new targeted therapy strategy (42, 45).

## Data availability statement

The raw data supporting the conclusions of this article will be made available by the authors, without undue reservation.

## Ethics statement

The studies involving human participants were reviewed and approved by IRCCS Humanitas Research Hospital ethics committee, study n° 1791, approved 06/06/2017. The patients/participants provided their written informed consent to participate in this study.

## Author contributions

Conception and design, RA and ML. Acquisition of data, VF, GL, MP, RC, PA, CS, AS, RH, NB, and PC. Analysis and interpretation of data, VF, ML, GS, IS, RA, and ML. Drafting of the manuscript, VF. Critical revision of the manuscript for important intellectual content, RA, ML, GS, and GL. Statistical analysis, VF. Obtaining funding, GG. Administrative, technical, or material support supervision, RA. All authors contributed to the article and approved the submitted version.

## Funding

This work was supported by the Ministero della Salute (Ricerca Finalizzata, grant number RF-2018-12367080) and by intramural funding (Fondazione Humanitas per la Ricerca).

## Acknowledgments

The authors would like to thank Nadia Lo Iacono as study coordinator. In addition, the authors thank and warmly remember Professor Stefano Duga, geneticist and co-investigator, who passed away of prostate cancer on October 10, 2021 at the age of 53 yr.

## Conflict of interest

The authors declare that the research was conducted in the absence of any commercial or financial relationships that could be construed as a potential conflict of interest.

## Publisher’s note

All claims expressed in this article are solely those of the authors and do not necessarily represent those of their affiliated organizations, or those of the publisher, the editors and the reviewers. Any product that may be evaluated in this article, or claim that may be made by its manufacturer, is not guaranteed or endorsed by the publisher.

## References

[1] Catalona WJ. Detection of organ-confined prostate cancer is increased through prostate-specific antigen–based screening. JAMA J Am Med Assoc (1993) 270:948. doi: 10.1001/jama.1993.03510080052031 7688438

[2] Cooperberg MR Lin DW Morgan TM Chapin BF Chen RC Eggener SE. Active surveillance: Very much “Preferred” for low-risk prostate cancer. J Urol (2022) 207:262–4. doi: 10.1097/JU.0000000000002341 34775795

[3] Maggi M Cowan JE Fasulo V Washington SL Lonergan PE Sciarra A. The long-term risks of metastases in men on active surveillance for early stage prostate cancer. J Urol (2020) 204:1222–8. doi: 10.1097/JU.0000000000001313 33157570

[4] Cooperberg MR Carroll PR Dall’Era MA Davies BJ Davis JW Eggener SE. The state of the science on prostate cancer biomarkers: The San Francisco consensus statement. Eur Urol (2019) 76:268–72. doi: 10.1016/j.eururo.2019.05.013 31128968

[5] Casale P Saita A Lazzeri M Lughezzani G Hurle R Fasulo V. p2PSA for predicting biochemical recurrence of prostate cancer earlier than total prostate-specific antigen after radical prostatectomy: An observational prospective cohort study. Miner Urol e Nefrol (2019) 71(3):273–9. doi: 10.23736/S0393-2249.19.03279-X 30700081

[6] Lazzeri M Haese A de la Taille A Palou Redorta J McNicholas T Lughezzani G. Serum isoform [–2]proPSA derivatives significantly improve prediction of prostate cancer at initial biopsy in a total PSA range of 2–10 ng/ml: A multicentric European study. Eur Urol (2013) 63:986–94. doi: 10.1016/j.eururo.2013.01.011 23375961

[7] de la Calle CM Fasulo V Cowan JE Lonergan PE Maggi M Gadzinski AJ. Clinical utility of 4Kscore ® , ExosomeDx^TM^ and magnetic resonance imaging for the early detection of high grade prostate cancer. J Urol (2021) 205:452–60. doi: 10.1097/JU.0000000000001361 32897802

[8] García-Perdomo HA Chaves MJ Osorio JC Sanchez A. Association between TMPRSS2:ERG fusion gene and the prostate cancer: Systematic review and meta-analysis. Cent Eur J Urol (2018) 71:410–9. doi: 10.5173/ceju.2018.1752 PMC6338815 30680235

[9] Park K Dalton JT Narayanan R Barbieri CE Hancock ML Bostwick DG. TMPRSS2:ERG gene fusion predicts subsequent detection of prostate cancer in patients with high-grade prostatic intraepithelial neoplasia. J Clin Oncol (2014) 32:206–11. doi: 10.1200/JCO.2013.49.8386 PMC3887478 24297949

[10] Leapman MS Nguyen HG Cooperberg MR. Clinical utility of biomarkers in localized prostate cancer. Curr Oncol Rep (2016) 18:30. doi: 10.1007/s11912-016-0513-1 27023445

[11] Punjani N Hayden RP Cai PY Wei JT Siddiqui J Dudley VL. PCA3 and Tmprss:Erg assessment in semen: Results of a phase I study. Fertil Steril (2020) 114:e389. doi: 10.1016/j.fertnstert.2020.08.1143

[12] Lazzeri M Colombo F Chiereghin C Buffi N Casale P Hurle R. PD06-10 liquid biopsy by prostate-derived tumor cells enriched from seminal fluid (Sf): The semen prostate cancer tumor elements (Spectre) project. J Urol (2018) 199:e154–e5. doi: 10.1016/j.juro.2018.02.430

[13] Donovan MJ Noerholm M Bentink S Belzer S Skog J O’Neill V. A molecular signature of PCA3 and ERG exosomal RNA from non-DRE urine is predictive of initial prostate biopsy result. Prostate Cancer Prostatic Dis (2015) 18:370–5. doi: 10.1038/pcan.2015.40 26345389

[14] Salami SS Schmidt F Laxman B Regan MM Rickman DS Scherr D. Combining urinary detection of TMPRSS2:ERG and PCA3 with serum PSA to predict diagnosis of prostate cancer. Urol Oncol Semin Orig Investig (2013) 31:566–71. doi: 10.1016/j.urolonc.2011.04.001 PMC3210917 21600800

[15] Sanda MG Feng Z Howard DH Tomlins SA Sokoll LJ Chan DW. Association between combined TMPRSS2:ERG and PCA3 RNA urinary testing and detection of aggressive prostate cancer. JAMA Oncol (2017) 3:1085. doi: 10.1001/jamaoncol.2017.0177 28520829 PMC5710334

[16] Pinto PA Chung PH Rastinehad AR Baccala AA Kruecker J Benjamin CJ. Magnetic resonance Imaging/Ultrasound fusion guided prostate biopsy improves cancer detection following transrectal ultrasound biopsy and correlates with multiparametric magnetic resonance imaging. J Urol (2011) 186:1281–5. doi: 10.1016/j.juro.2011.05.078 PMC3193933 21849184

[17] Chu CE Cowan JE Lonergan PE Washington SL Fasulo V de la Calle CM. Diagnostic accuracy and prognostic value of serial prostate multiparametric magnetic resonance imaging in men on active surveillance for prostate cancer. Eur Urol Oncol (2021) 19:S2588-9311(20)30203-0. doi: 10.1016/j.euo.2020.11.007 33483265

[18] Hamoen EHJ de Rooij M Witjes JA Barentsz JO Rovers MM. Use of the prostate imaging reporting and data system (PI-RADS) for prostate cancer detection with multiparametric magnetic resonance imaging: A diagnostic meta-analysis. Eur Urol (2015) 67:1112–21. doi: 10.1016/j.eururo.2014.10.033 25466942

[19] Weinreb JC Barentsz JO Choyke PL Cornud F Haider MA Macura KJ. PI-RADS prostate imaging – reporting and data system: 2015, version 2. Eur Urol (2016) 69:16–40. doi: 10.1016/j.eururo.2015.08.052 26427566 PMC6467207

[20] Mottet N Bellmunt J Briers E Bolla M Bourke L Cornford P. EAU guidelines. edn. presented at the EAU annual congress Milan 2021 (2021). Available at: https://uroweb.org/guideline/prostate-cancer/.

[21] Lughezzani G Saita A Lazzeri M Paciotti M Maffei D Lista G. Comparison of the diagnostic accuracy of micro-ultrasound and magnetic resonance Imaging/Ultrasound fusion targeted biopsies for the diagnosis of clinically significant prostate cancer. Eur Urol Oncol (2019) 2:329–32. doi: 10.1016/j.euo.2018.10.001 31200848

[22] Lopci E Lughezzani G Castello A Colombo P Casale P Saita A. PSMA-PET and micro-ultrasound potential in the diagnostic pathway of prostate cancer. Clin Transl Oncol (2021) 23:172–8. doi: 10.1007/s12094-020-02384-w 32447644

[23] Lopci E Saita A Lazzeri M Lughezzani G Colombo P Buffi NM. 68 Ga-PSMA positron emission Tomography/Computerized tomography for primary diagnosis of prostate cancer in men with contraindications to or negative multiparametric magnetic resonance imaging: A prospective observational study. J Urol (2018) 200:95–103. doi: 10.1016/j.juro.2018.01.079 29409824

[24] Ghai S Eure G Fradet V Hyndman ME McGrath T Wodlinger B. Assessing cancer risk on novel 29 MHz micro-ultrasound images of the prostate: Creation of the micro-ultrasound protocol for prostate risk identification. J Urol (2016) 196:562–9. doi: 10.1016/j.juro.2015.12.093 26791931

[25] Lopci E Lughezzani G Castello A Saita A Colombo P Hurle R. Prospective evaluation of 68Ga-labeled prostate-specific membrane antigen ligand positron emission Tomography/Computed tomography in primary prostate cancer diagnosis. Eur Urol Focus (2020) 7(4):764–71. doi: 10.1016/j.euf.2020.03.004 32312701

[26] Mortezavi A Palsdottir T Eklund M Chellappa V Murugan SK Saba K. Head-to-head comparison of conventional, and image- and biomarker-based prostate cancer risk calculators. Eur Urol Focus (2021) 7:546–53. doi: 10.1016/j.euf.2020.05.002 32451315

[27] Petrovics G Liu A Shaheduzzaman S Furasato B Sun C Chen Y. Frequent overexpression of ETS-related gene-1 (ERG1) in prostate cancer transcriptome. Oncogene (2005) 24:3847–52. doi: 10.1038/sj.onc.1208518 15750627

[28] Tomlins SA Rhodes DR Perner S Dhanasekaran SM Mehra R Sun X-W. Recurrent fusion of TMPRSS2 and ETS transcription factor genes in prostate cancer. Science (2005) 310:644–8. doi: 10.1126/science.1117679 16254181

[29] Kong D-P Chen R Zhang C-L Zhang W Xiao G-A Wang F-B. Prevalence and clinical application of TMPRSS2-ERG fusion in Asian prostate cancer patients: a large-sample study in Chinese people and a systematic review. Asian J Androl (2020) 22:200. doi: 10.4103/aja.aja_45_19 31210145 PMC7155806

[30] Prensner JR Rubin MA Wei JT Chinnaiyan AM. Beyond PSA: the next generation of prostate cancer biomarkers. Sci Transl Med (2012) 4:127rv3. doi: 10.1126/scitranslmed.3003180 PMC3799996 22461644

[31] Mosquera J-M Mehra R Regan MM Perner S Genega EM Bueti G. Prevalence of TMPRSS2-ERG fusion prostate cancer among men undergoing prostate biopsy in the united states. Clin Cancer Res (2009) 15:4706–11. doi: 10.1158/1078-0432.CCR-08-2927 PMC3717524 19584163

[32] Zhou CK Young D Yeboah ED Coburn SB Tettey Y Biritwum RB. TMPRSS2:ERG gene fusions in prostate cancer of West African men and a meta-analysis of racial differences. Am J Epidemiol (2017) 186:1352–61. doi: 10.1093/aje/kwx235 PMC5860576 28633309

[33] Laxman B Tomlins SA Mehra R Morris DS Wang L Helgeson BE. Noninvasive detection of TMPRSS2:ERG fusion transcripts in the urine of men with prostate cancer. Neoplasia (2006) 8:885–8. doi: 10.1593/neo.06625 PMC1715928 17059688

[34] Hessels D Smit FP Verhaegh GW Witjes JA Cornel EB Schalken JA. Detection of TMPRSS2-ERG fusion transcripts and prostate cancer antigen 3 in urinary sediments may improve diagnosis of prostate cancer. Clin Cancer Res (2007) 13:5103–8. doi: 10.1158/1078-0432.CCR-07-0700 17785564

[35] Osses D Roobol M Schoots I. Prediction medicine: Biomarkers, risk calculators and magnetic resonance imaging as risk stratification tools in prostate cancer diagnosis. Int J Mol Sci (2019) 20:1637. doi: 10.3390/ijms20071637 PMC6480079 30986955

[36] Sanguedolce F Cormio A Brunelli M D’Amuri A Carrieri G Bufo P. Urine TMPRSS2: ERG fusion transcript as a biomarker for prostate cancer: Literature review. Clin Genitourin Cancer (2016) 14:117–21. doi: 10.1016/j.clgc.2015.12.001 26774207

[37] Stephan C Jung K Semjonow A Schulze-Forster K Cammann H Hu X. Comparative assessment of urinary prostate cancer antigen 3 and TMPRSS2:ERG gene fusion with the serum [–2]Proprostate-Specific antigen–based prostate health index for detection of prostate cancer. Clin Chem (2013) 59:280–8. doi: 10.1373/clinchem.2012.195560 23213079

[38] Kurhanewicz J Vigneron D Carroll P Coakley F. Multiparametric magnetic resonance imaging in prostate cancer: Present and future. Curr Opin Urol (2008) 18:71–7. doi: 10.1097/MOU.0b013e3282f19d01 PMC2804482 18090494

[39] Mottet N Bastian P Bellmunt J van den Bergh R Bolla M van Casteren N. EAU - EANM - ESTRO - ESUR - SIOG: Guidelines on prostate cancer. Arnhem, The Netherlands: EAU Guidelines Office (2020). Available at: https://uroweb.org/guideline/prostate-cancer/.

[40] Lughezzani G Maffei D Saita A Paciotti M Diana P Buffi NM. Diagnostic accuracy of microultrasound in patients with a suspicion of prostate cancer at magnetic resonance imaging: A single-institutional prospective study. Eur Urol Focus (2021) 7:1019–26. doi: 10.1016/j.euf.2020.09.013 33069624

[41] Perner S Mosquera J-M Demichelis F Hofer MD Paris PL Simko J. TMPRSS2-ERG fusion prostate cancer: An early molecular event associated with invasion. Am J Surg Pathol (2007) 31:882–8. doi: 10.1097/01.pas.0000213424.38503.aa 17527075

[42] Kohaar I Li Q Chen Y Ravindranath L Young D Ali A. Association of germline genetic variants with TMPRSS2-ERG fusion status in prostate cancer. Oncotarget (2020) 11:1321–33. doi: 10.18632/oncotarget.27534 PMC7170497 32341752

[43] Bancroft EK Page EC Brook MN Thomas S Taylor N Pope J. A prospective prostate cancer screening programme for men with pathogenic variants in mismatch repair genes (IMPACT): Initial results from an international prospective study. Lancet Oncol (2021) 22:1618–31. doi: 10.1016/S1470-2045(21)00522-2 PMC8576477 34678156

[44] Fasulo V Zuradelli M Lazzeri M. Re: A prospective prostate cancer screening programme for men with pathogenic variants in mismatch repair genes (IMPACT): Initial results from an international prospective study. Eur Urol (2021) 81(2):216–8. doi: 10.1016/j.eururo.2021.11.030 34895922

[45] Lorenzin F Demichelis F. Past, current, and future strategies to target ERG fusion-positive prostate cancer. Cancers (Basel) (2022) 14:1118. doi: 10.3390/cancers14051118 35267426 PMC8909394

